# Effect of zonisamide on sleep and rapid eye movement sleep behavioral disorders in patients with Parkinson’s disease: A randomized control trial

**DOI:** 10.1016/j.prdoa.2024.100285

**Published:** 2024-11-22

**Authors:** Hiroshi Kataoka, Masahiro Isogawa, Hitoki Nanaura, Hiroyuki Kurakami, Miyoko Hasebe, Kaoru Kinugawa, Takao Kiriyama, Tesseki Izumi, Masato Kasahara, Kazuma Sugie

**Affiliations:** a Department of Neurology, Nara Medical University, Kashihara, Nara, Japan; b Institute for Clinical and Translational Science, Nara Medical University Hospital, Kashihara, Nara, Japan

**Keywords:** Parkinson, Zonisamide, Sleep, REM sleep behavioral disorders, Clinical trial

## Abstract

**Introduction:**

Zonisamide is a medication developed in Japan that is effective for motor symptoms and wearing off in Parkinson’s disease (PD). Zonisamide has properties that may improve sleep disorders. The aim of this study is to verify the safety and efficacy of zonisamide for sleep disorders and rapid eye movement (REM) sleep behavioral disorders (RBD) using a mobile two-channel electroencephalography /electrooculography recording system in patients with PD.

**Methods:**

The present study is a single-blind randomized placebo-controlled trial. The subjects in the treatment group took zonisamide (25 mg per day) before bedtime. The primary outcome was sleep efficiency. The secondary endpoints were assessed as followed; objective outcomes of TST*,* WASO, SOL, REM sleep/non-REM sleep ratio, deep sleep (N3) time, ratio of RWA to total REM sleep epochs, and subjective outcomes of the PDSS-2, Pittsburgh sleep questionnaire, and RBDSQ.

**Results:**

Between the zonisamide and placebo groups, no significant differences were found in the primary outcome and secondary outcomes.

**Conclusions:**

The objective and subjective sleep metrics in this clinical trial did not significantly demonstrate zonisamide efficacy for sleep disorder in patients with PD. Although not significant, improvement in WASO and SOL was observed when zonisamide was compared with the placebo.

## Highlights

• The present study is a randomized placebo-controlled trial in Parkinson’s disease.
• This study is to verify the safety and efficacy of zonisamide for sleep disorders.
• This study also objectively evaluate REM sleep behavioral disorders (RBD).
• Mobile electroencephalography /electrooculography recording system was used.

## 1 Introduction

Zonisamide (1,2‐benzisoxazole‐3‐methanesulfonamide) is a medication developed in Japan that is effective for improving motor symptoms and reducing wearing off in patients with PD. It is safe with a very low incidence of dyskinesia and psychiatric symptoms, such as hallucinations [1], [2], [3]. However, owing to the limited numbers of randomized clinical trials, the efficacy of zonisamide in treating non-motor symptoms remains unclear. Moreover, the efficacy of dopaminergic medications for the treatment of non-motor symptoms of PD remains debatable [4], [5].

Sleep disorders are a frequent non-motor symptom and an early indicator of PD [6], [7]. For instance, sleep fragmentation, with an estimated rate of 74–88 % [8], [9], is closely associated with many aspects of PD pathology [10]. Meanwhile, sleep is known to contribute positively to dopamine receptor regulation and dopamine storage [10], [11], [12]. Furthermore, the levels of dopamine in the synaptic cleft increase with good sleep quality [13]. Therefore, the pathophysiological importance of sleep in PD is garnering increased attention. However, very few clinical trials have been carried out so far on the efficacy of dopaminergic drugs for the treatment of sleep disorders in PD. For instance, rotigotine [14] and chronic-release levodopa/carbidopa [15] improve some aspects of sleep; however, pergolide worsens sleep efficiency and sleep fragmentation [16]. Nonetheless, evidence supporting the efficacy of dopaminergic medications for sleep disorders is quite limited, particularly in randomized placebo-controlled studies. Zonisamide has a long half-life with a low potential for interacting with other medications and can improve nocturnal motor symptoms, leading to better sleep. Furthermore, zonisamide indirectly reduces glutamatergic activity and enhances GABAergic activity [17]; moreover, it inhibits monoamine oxidase (MAO) activity, which mediates its effects on sleep quality [18]. A *meta*-analysis showed that in patients with PD, zonisamide may increase the probability of somnolence [19].

Rapid eye movement (REM) sleep behavioral disorders (RBDs) have also become a particularly important feature of PD pathology. Idiopathic RBD is recognized as the prodromal stage of PD, and PD with RBD shows faster progression and more cognitive decline than PD alone [20], [21]. RBD is related to degeneration of the brain stem sleep regulatory center; therefore, insomnia and RBD are more closely associated than was realized before [22]. Melatonin and clonazepam have been widely used to treat RBD symptoms, while some anti-Parkinsonian medications such as rotigotine [23], rasagiline [24], and safinamide [25] have been shown to improve objective polysomnographic sleep measures. Furthermore, several randomized placebo-controlled trials have documented conflicting findings regarding the efficacy of RBD [26]. In 2012, our group reported that both vivid nightmares and dream-enacting behavior were dramatically resolved in a patient with PD treated with zonisamide [27]. In 2015, zonisamide monotherapy was administered to 10 patients with de novo PD, contributing to the inhibition of nocturnal RBD symptoms in three patients with a prior history of RBD [28]. These findings warrant further research on whether zonisamide might contribute to the potential improvement in RBD.

Because most RBD symptoms go undetected and patients are unaware of them, newly developed at-home polysomnography that can detect RBD in clinical trials was inevitable [26]. Polysomnography (PSG) requires patients to stay overnight in a hospital or sleep center; however, the two-channel electroencephalography (EEG)/electrooculography (EOG) recording system can measure natural sleep at home and diagnose RBD in an outpatient care setting. Previously, our team performed a validation study comparing a two-channel EEG/EOG recording system with polysomnography in patients with PD and found that the estimated sleep variables, including REM sleep without atonia (RWA), correlated well between both systems [29]. The aim of this study was to assess the safety and efficacy of zonisamide for the treatment of sleep disorders and RBD using a mobile two-channel EEG/EOG recording system in patients with PD.

## 2 Materials and Methods

### 2.1 Study design and participants

ZEAL (Zonisamide for the Efficacy of Sleep Abnormality in Parkinson's Disease) study is a randomized, single-blind, placebo-controlled trial conducted at Nara Medical University Hospital in Japan that investigated the efficacy of zonisamide for sleep disorders in patients with PD. The protocol for this study has been previously published [30]. Briefly, 25 patients showed the decline in striatal uptake on the dopamine transporter in single photon emission computed tomography and 37 patients showed the decrease in myocardial uptake of I-123 metaiodobenzylguanidine (MIBG) on myocardial scintigraphy. Apart from the decline in striatal uptake on the dopamine transporter in single photon emission computed tomography, all patients with PD aged ≥ 41 years who met the International Parkinson and Movement Disorder Society (MDS) diagnostic criteria for PD [31] were included in the current study. All patient had a Mini-Mental State Examination score of ≥ 22 and reported at least one of the following sleep issue: (1) a response of “Sometimes,” “Almost none,” or “Nothing” on item 1 (question: “Did you sleep well last week?”) on the PD Sleep Scale (PDSS)-2 Japanese version [32]; (2) a response of “Sometimes,” “Many,” or “So much” on item 2 (question: “Did you have a bad day at night?”) on the PDSS-2 Japanese version [32]; or (3) a score of ≥ 5 on the 10-item no/yes Sleep Behavior Disorder Screening questionnaire Japanese version (RBDSQ), with a maximum score of 13 [33]. All patients were managed as outpatients. The patients who had been treated with zonisamide within 3 months prior to obtaining informed consent; had a history of brain surgery or other organic cerebral disorders; were taking both MAO-B inhibitors and tricyclic antidepressants; had comorbidities such as severe dyskinesia; and a significant history of illness such as malignant syndrome were excluded [30]. The eligible patients provided written consent to participate in this trial. The protocol for this trial was approved by the Research Ethics Committee of Nara Medical University and was registered with the Japan Registry of Clinical Trials (jRCTs051200160).

### 2.2 Randomization and blinding

This was a single-blind study; however, subject allocation was randomized by an independent allocation manager via computer-generated block randomization. The patients were randomly assigned to zonisamide treatment and control groups in a 1:1 ratio.

### 2.3 Procedure and schedule

Patients in the treatment group received zonisamide (25 mg/day) before bedtime for 28 days. Two weeks prior to enrollment, at the initiation of the intervention, and during the clinical trial period, participants maintained stable regimens and a constant dose of anti-PD medications and other drugs, such as sleep stabilizers [30]. During this period, introducing new dosages of these medications was prohibited. Regarding the medications used to treat RBD, the included patients did not receive melatonin. If the patient was taking clonazepam, the drug was discontinued at least 3 months prior to enrollment. The outcome assessments were performed at 14 and 28 days after randomization.

### 2.4 Primary and secondary endpoints

The primary endpoint was sleep efficiency (SE; %), recorded using a self-applicable and affordable two-channel EEG/EOG recording system (SleepGraph; Medical Device Certification Number: 231AHBZX00001000). The secondary endpoints for efficacy were assessed as follows: objective outcomes of total sleep time (TST)*,* wake time after sleep onset (WASO), sleep onset latency (SOL), REM sleep/non-REM sleep ratio, deep sleep (N3) time, ratio of RWA to total REM sleep epochs (%), and subjective outcomes of the PDSS-2, Pittsburgh sleep questionnaire, and RBDSQ. The sleep index, recorded using the EEG/EOG recording system, was obtained for two consecutive nights within two days prior to the intervention. Subsequently, it was reassessed one night following the completion of the 28-day administration of zonisamide. The PDSS-2, Pittsburgh sleep questionnaire, and RBDSQ data were collected at baseline and at 14 and 28 days after zonisamide administration. Other clinical evaluation tools, such as the Movement Disorder Society Unified Parkinson’s Disease Rating Scale (MDS-UPDRS) Part 3 and Part 4 [34] were also used.

### 2.5 Statistical analysis

Data are described as mean ± standard deviation (SD) when normally distributed or as median with the range when non-normally distributed. The mean and median values between groups were analyzed using the *t*-test and Wilcoxon rank-sum test, respectively. Categorical data were analyzed using the χ2 or Fisher's exact tests. The patients excluded from the full analysis set (FAS) were those who did not meet the eligibility requirements or had no data after allocation. Regarding the primary outcome, the amount of change in SE after the 28-day administration between the zonisamide treatment and placebo groups was compared. As for the secondary endpoint, the change in the ratio of RWA to total REM sleep epochs, TST, WASO, SOL, ratio of REM/NREM sleep, and deep sleep (N3) time was compared between the two groups. The alteration of each score of the PDSS-2, Pittsburgh sleep questionnaire, or RBDSQ was compared in each group using paired *t*-test or Wilcoxon signed rank-sum test. The population on zonisamide is referred to as the safety analysis set (SAS). Per Protocol Set (PPS) is the portion of the FAS that strictly conforms to the study protocol. P-values were two-sided with values < 0.05 regarded as statistically significant. All statistical analyses were performed using SAS version 9.4 (SAS Institute Inc., Cary, NC, USA).

## 3 Results

**Fig. 1:**
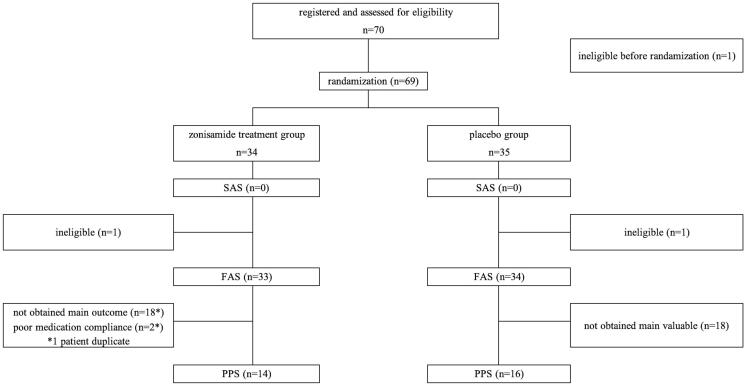
Flow diagrams of patient’s recruitment and randomization. SAS: Safety Analysis Set, FAS: full analysis set., PPS: Per Protocol Set.

Twenty-eight days following randomization, FAS numbers in the zonisamide treatment and placebo groups were 33 and 34 patients, respectively (Fig. 1). Thirty patients (PPS; zonisamide n = 14, placebo n = 16) out of those with FAS had pre- and post-intervention data on the portable EEG/EOG recording system. Additionally, there were 10 dropouts at baseline, and 14 dropouts at 28 days in the zonisamide group, while the placebo group had 12 dropouts at baseline and 12 dropouts at 28 days after intervention on the portable EEG/EOG recording system. EEG/EOG recording alterations were analyzed in 15 patients out of FAS numbers who had both pre- and post-intervention data; there were 18 dropouts in both zonisamide and placebo groups.

**Fig. 2:**
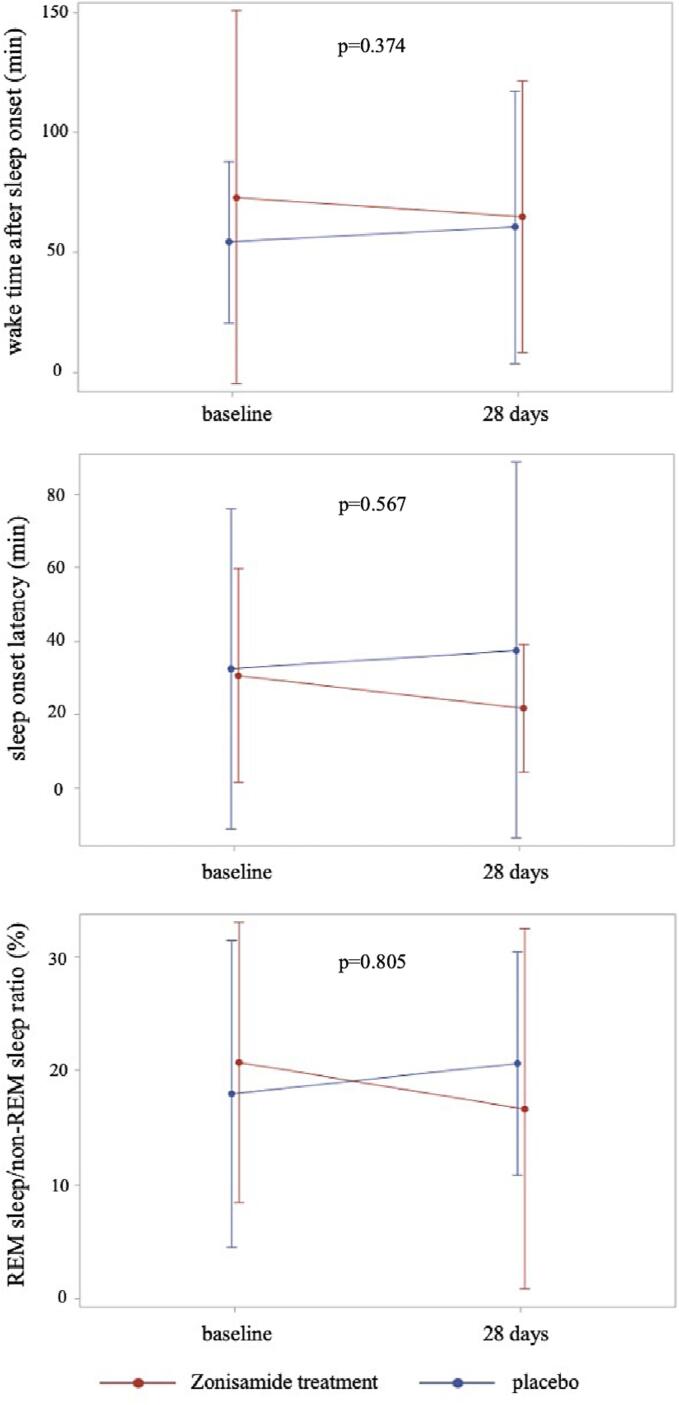
Change of objective sleep indicators on the two-channel electroencephalography /electrooculography at-home recording system following zonisamide treatment. After treatment with zonisamide, the zonisamide group showed a decrease in the wake time after sleep onset (WASO) (upper panel), sleep onset latency (SOL) (middle panel), and REM sleep/non-REM sleep ratio (lower panel); however, these parameters increased in the placebo group.

The following sleep-related variables were not significantly different between the zonisamide treatment and placebo groups at baseline: psychosis, anxiety on the MDS-Non Motor Rating Scale, Parkinson's Fatigue Scale [35], Beck Depression Inventory in addition to Hoehn-Yahr stage, MDS-UPDRS parts 3 and 4, and PD duration (Table 1). Twenty-eight days after the randomization, no significant differences were found between the zonisamide treatment and placebo groups in the SE of the primary outcome; and TST, WASO, SOL, and REM sleep/non-REM sleep ratio, deep sleep (N3) time of the secondary outcome (Table 2). RWA to total REM sleep epochs before and after twenty-eight days was not significantly differed in zonisamide (median 8.90, range 0 to 51 and 5.70, 0 to 71.4, respectively, p = 0.916) and placebo group (4.25, 0 to 84.3 and 8.80, 0 to 61.3, respectively, p = 0.935). At 28 days, WASO and SOL in the zonisamide treatment group were shorter than those in the placebo group but not significantly different, with mean differences for WASO and SOL of 20.42 and 5.86 min, respectively. Additionally, the change in the ratio of REM to non-REM sleep in the zonisamide treatment group was larger than that in the placebo group (Fig. 2). Compared with the placebo group, the zonisamide group's PDSS-2 and Pittsburgh sleep questionnaire scores did not show a statistically significant change at 14 and 28 days following randomization (Table 3). The RBDSQ score in the zonisamide treatment group was significantly lower at 14 days following randomization (p = 0.035) but increased again at 28 days. The zonisamide treatment group showed a significant drop in the MDS-UPDRS Part 3 score (p < 0.001), whereas no significant difference was found in the MDS-UPDRS Part 4 scores (Supplemental Table 1). In terms of safety, the zonisamide treatment group experienced two side effects which necessitated the halting of zonisamide: excessive sleepiness and urine incontinence. Three minor side effects not causally related to zonisamide included falls due to the primary disease and nausea in zonisamide group, and swelling of the left dorsum of the foot in the placebo group. The prevalence of these side effects showed no significant difference between the zonisamide and placebo groups (supplemental Table 2).

**Table 1:** Baseline characteristics in the zonisamide treatment and placebo groups.

	zonisamide group n = 33	placebo n = 34	p
age, mean, years	72.8 ± 7.6	71.9 ± 8.3	0.632
men, n	19 (57.6)	18 (52.9)	0.703 §
height (cm), mean	160.14 ± 7.78	157.81 ± 9.48	0.227
weight (kg), mean	56.15 ± 9.8	55.28 ± 12.84	0.759
disease duration (months), median	112.0, 60 to 408	108.0, 60 to 276	0.342 †
presence of comorbidities associated with PD	15 (45.5)	16 (47.1)	0.895
Mini-Mental State Examination, median	29.0, 26 to 30	29.0, 23 to 30	0.845 †
Hoehn-Yahr stage Ⅰ, n	3 (9.1)	4 (11.8)	0.943 ‡
Ⅱ	18 (54.5)	16 (47.1)	
Ⅲ	7 (21.2)	7 (20.6)	
Ⅳ	5 (15.2)	7 (20.6)	
MDS-UPDRS part 3, mean	27.9 ± 15.0	23.9 ± 13.1	0.25
MDS-UPDRS part 4, median	4.0, 0 to 15.0	4.0, 0 to 17.0	0.84 †
PDSS-2, mean	22.9 ± 8.3	23.2 ± 8.6	0.87
motor symptoms at night	6.4 ± 3.6	6.0 ± 3.7	0.718
PD symptoms at night	5.7 ± 3.6	5.9 ± 4.0	0.83
disturbed sleep	10.8 ± 3.0	11.3 ± 2.8	0.514
RBDSQ, mean	5.7 ± 3.1	5.6 ± 3.1	0.924
Pittsburg sleep questionnaire, mean	8.5 ± 4.0	8.8 ± 3.3	0.737
Beck Depression Inventory, mean	13.8 ± 7.5	16.0 ± 9.9	0.322
Parkinson’s Fatigue scale	47.9 ± 15.7	53.7 ± 15.9	0.151
MDS-Non motor rating scale			
psychosis, median	0, 0 to 10	0, 0 to 18	0.795 †
anxiety, median	4.0, 0 to 22	5.5, 0 to 20	0.6 †

PD: Parkinson's disease, PDSS: Parkinson’s Disease Sleep Scale-2 Japanese version,

RBDSQ: Sleep Behavior Disorder Screening questionnaire Japanese version,

MDS-UPDRS: Movement Disorder Society Revision of the Unified PD Rating Scale,

data are reported as mean (standard deviation), median (range), or number (%),

† Wilcoxon's rank sum test, § χ2 test, ‡ Fisher's exact test.

**Table 2:** Change of objective sleep parameters between the zonisamide treatment and placebo groups on the full analysis set (FAS).

	zonisamide treatment			placebo			
	baseline (n = 23)	28 days (n = 19)	quantity of alteration (n = 15) ^a^	baseline (n = 22)	28 days (n = 22)	quantity of alteration (n = 16) ^a^	p*
dropouts number (n)	10	14	18	12	12	18	
primary outcome							
sleep efficiency (%)	82.81 ± 16.33	83.59 ± 14.16	0.21 ± 12.35	86.45 ± 8.38	85.19 ± 11.36	−2.45 ± 12.53	0.556
secondary outcomes objective outcome							
Total sleep time (TST) (min)	321.87 ± 84.89	313.84 ± 105.23	−11.77 ± 72.10	348.30 ± 103.96	339.50 ± 115.68	−15.28 ± 81.81	0.9
Wake time after sleep onset (WASO) (min)	72.96 ± 77.79	64.71 ± 56.51	−8.73 ± 60.65	54.14 ± 33.78	60.67 ± 56.81	11.69 ± 65.02	0.374
Sleep onset latency (SOL) (min).	30.70 ± 29.13	21.84 ± 17.43	−2.17 ± 23.59	32.57 ± 43.61	37.61 ± 51.24	3.69 ± 31.82	0.567
REM sleep/non-REM sleep ratio (%)	20.75 ± 12.27	16.66 ± 15.78	−1.7 ± 17.18	17.98 ± 13.44	20.62 ± 9.78	−0.42 ± 11.09	0.805
Deep sleep (N3) time (min)	69.30 ± 30.03	66.50 ± 38.25	−8.90 ± 33.08	68.09 ± 29.84	55.23 ± 27.63	−13.91 ± 37.84	0.699

* comparison of quantity of alteration between zonisamide treatment and placebo groups.

a: the quantity of alteration of the EEG/EOG recording system was analyzed patients who had both pre- and post-intervention data.

**Table 3:** Change of subjective sleep scores between the zonisamide treatment and placebo groups in the full analysis set (FAS).

	zonisamide treatment							placebo						
	baseline	14 days	28 days	quantity of alteration (14 days)	p*	quantity of alteration (28 days)	p*	baseline	14 days	28 days	quantity of alteration (14 days)	p*	quantity of alteration (28 days)	p*
PDSS-2	22.9 ± 8.3 (n = 33) ^a^	21.7 ± 8.7 (n = 32) ^a^	22.0 ± 7.8 (n = 33) ^a^	−1.2 ± 6.0 (n = 32) ^b^	0.263	−0.8 ± 6.8 (n = 33) ^b^	0.478	23.2 ± 8.6 (n = 31) ^a^	23.3 ± 10.0 (n = 34) ^a^	19.7 ± 7.6 (n = 33) ^a^	−0.1 ± 6.6 (n = 31) ^b^	0.914	−3.7 ± 6.7 (n = 31) ^b^	0.005
Pittsburg Sleep Questionnaire	8.5 ± 4.0 (n = 32) ^a^	8.3 ± 3.8 (n = 32) ^a^	8.2 ± 3.7 (n = 33) ^a^	−0.3 ± 2.5 (n = 32) ^b^	0.526	−0.4 ± 2.7 (n = 32) ^b^	0.436	8.8 ± 3.3 (n = 32) ^a^	8.9 ± 3.9 (n = 32) ^a^	8.5 ± 3.6 (n = 31) ^a^	0 ± 2.9 (n = 31) ^b^	0.951	−0.3 ± 2.5 (n = 30) ^b^	0.513
RBDSQ	5.7 ± 3.1 (n = 32) ^a^	4.4 ± 2.5 (n = 32) ^a^	5.3 ± 3.3 (n = 31) ^a^	−1.2 ± 3.1 (n = 31) ^b^	0.035*	−0.4 ± 2.2 (n = 30) ^b^	0.295	5.6 ± 3.1 (n = 31) ^a^	5.2 ± 2.8 (n = 31) ^a^	4.5 ± 2.8 (n = 32) ^a^	−0.4 ± 2.2 (n = 28) ^b^	0.357	−1.1 ± 2.6 (n = 30) ^b^	0.032*

PDSS-2:Parkinson’s Disease Sleep Scale-2 Japanese version, RBDSQ: Sleep Behavior Disorder Screening questionnaire Japanese version, data are reported as mean (standard deviation), *p < 0.05, * comparison of quantity of alteration in each group between baseline and 14 or 28 days, a; total number of respondents to all questions at baseline, 14 days, and 28 days following the intervention. b: the quantity of alteration of three subjective sleep scores was analyzed patients who had both pre- and post-intervention data.

## 4 Discussion

In the current study, zonisamide did not significantly alter objective and subjective sleep parameters in patients with PD; however, some observations suggest that zonisamide may reduce sleep disorders in these patients.

Sleep fragmentation is the most common complaint during night-time sleep in patients with PD [8]. Patients with sleep fragmentation have more midbrain neuronal loss and Lewy bodies than those without sleep fragmentation, suggesting that this symptom accelerates PD pathology [10]. Furthermore, sleep fragmentation can contribute to daytime sleepiness [36] and reduced motor mobility in the morning, while improved sleep quality reduces the severity of morning off episodes [37]. Medications for treating PD can have various positive and negative effects on sleep fragmentation. In 2023, one open-label study evaluated the efficacy of zonisamide (timing of intake was not available) for objective sleep assessments in six patients with PD [38]. The first polysomnographic 12-week study revealed no efficacy for WASO, SE, or TST. In the present study, despite lacking statistical significance, WASO in the zonisamide treatment group decreased following the four-week treatment, whereas it increased in the placebo group. This finding suggests that zonisamide may decrease sleep fragmentation; the lack of significance may be explained by the small sample size and statistical power. Reduced percentages of time spent in sleep stages N2 and N3 as well as an increase in the percentage of time spent in sleep stage N1 are characteristic polysomnographic findings of PD [39]. In the current study, the REM sleep/non-REM sleep ratio decreased but no change was found in deep sleep (N3) time after zonisamide treatment, suggesting that the decline in the proportion of time in stage N2, a characteristic of PD, may be slightly mitigated by zonisamide. A previous polysomnographic study of six patients with PD reported that in addition to the non-statistical proportion of time spent in stage N3 sleep and the decrease in the percentage of time spent in stage N1 sleep, the proportion of time spent in sleep stage N2 increased after zonisamide treatment [38]. Additionally, SOL decreased; however, not significantly. Furthermore, a low dose of zonisamide had a safe adverse effect profile, with only two patients discontinuing treatment due to adverse effects, such as urinary incontinence and excessive sleepiness.

RBD is an important symptom in the prodromal stage of α-synucleinopathies, particularly in PD and after the development of motor symptoms, and its relevance is recently growing. Neurodegenerative processes in the brainstem, which is located near the sleep regulatory center and is strongly linked to sleep, are among the underlying pathophysiological mechanisms of PD. Nevertheless, only a few randomized placebo-controlled studies have shown reduced RBD in patients with PD, and contradictory findings have been documented [26]. Unfortunately, the current study did not report the efficacy of zonisamide for the treatment of RBD symptoms. While subjective RBDSQ declined considerably two weeks after zonisamide administration, at four weeks, the score approached baseline. Conflicting results regarding subjective improvements in RBD symptoms and objective polysomnographic parameter deterioration have been reported [26]. For instance, one crossover polysomnographic study using safinamide showed an improvement in some sub-items of Honkon’s RBDSQ; however, no decline in RWA was observed on polysomnography [25].

The finding of the subjective improvement in the RBD score at two weeks after randomization indicates that zonisamide treatment leads to an early reduction in RBD symptoms. A case of PD in which nightmares and violent behavior were substantially resolved shortly after starting zonisamide treatment has been reported [27]. Previously, the PDSS-2 score decreased at 1 and 2 months after zonisamide treatment; however, the score at 3 months was higher than that at 2 months, although the score between baseline and 3 months differed significantly [17]. These findings suggests that zonisamide may have had a relatively short effect. A larger study evaluating RWA using an objective at-home recording system immediately following zonisamide treatment is required.

No evidence of improvement in the PDSS-2 total scores on zonisamide treatment was found in the current study. A previous open-label study of 20 patients reported a reduction in the PDSS-2 total scores after zonisamide treatment, especially in the tremor group [17]. Our study did not evaluate the PDSS score in PD patients with tremors (defined as those with a score of 1 or higher on sub-items 3.15, 3.16, and 3.17 of the MDS-UPDRS), the population which had previously shown a reduction in PDSS-2 total scores in the study [17]. Additionally, patients in the present study were administered 25 mg, not 50 mg of zonisamide unlike in the study by Suzuki et al [17]. The incidence of somnolence as an adverse effect is considerably higher in patients receiving 50 mg compared to 25 mg [1], [2]. The sleep evaluation results in this study could therefore be influenced by somnolence. To minimize this risk, we selected a 25 mg dose.

Regarding the sample size, the protocol required a minimum of 15 patients per group to compare the objective sleep measures on the two-channel EEG/EOG recording system between both groups. However, we estimated that a need for 35 patients per group due to the challenges faced by patients with PD in attaching this recording system [30]. The patients were unable to obtain complete RWA data before and after randomization in the two-channel EEG/EOG recording system, and some patients who had evident RWA prior to randomization were unable to obtain RWA data after the treatment. Consequently, the statistical power might have been insufficient to achieve statistical significance. Further, a power analysis, which is generally calculated based on the results of past pilot studies and retrospective studies, was not performed to estimate the sample size in this study, as there was no data on EEG/EOG recording system for the patients with PD before. The results of the current study, including the missing data, will make it possible to calculate more reliable sample sizes in the future. One of the challenges of using this equipment for patients with PD is the installation of an EEG device at home. Missing data for the EEG/EOG recording system other than PPS were included in the analysis as FAS. Compared to the EEG/EOG recording system, which was used only once following the intervention, there were fewer missing data for the EEG/EOG recording system, which was used twice before administration. Future research employing this system may reduce dropout rates by increasing the number of pre- and post-intervention measurements, particularly by performing two post-intervention measurements. This approach could increase the number of PPS evaluations and potentially impact the primary and secondary outcome results.

To the best of our knowledge, this zonisamide-based randomized clinical trial is the first to assess RBD in addition to other sleep disturbances in patients with PD. As for the limitations, the results of the current study may have been limited by the modest sample size. An easily accessible at-home device for PD that measures several aspects of sleep, such as the RWA, is anticipated. Because the number of PPS was too small, PPS data from before and after treatment was not examined; therefore, the analysis focused on FAS.

In conclusion, zonisamide at a dose of 25 mg/day before bedtime is safe and effective in alleviating motor complaints in patients with PD. However, the objective and subjective sleep metrics in this clinical trial did not demonstrate a significant efficacy for zonisamide in the treatment of RBD symptoms in patients with PD. Although not significant, improvements in WASO and SOL were observed when zonisamide was compared with the placebo. Larger longitudinal studies including different doses of zonisamide are required to demonstrate its efficacy on RBD in patients with PD.

## Ethical approval

The study protocol was approved by the Nara Medical University Certified Review Board, 2020 and it has been registered with Japan Registry of Clinical Trials (jRCTs051200160).

## CRediT authorship contribution statement

**Hiroshi Kataoka:** Writing – review & editing, Writing – original draft, Supervision, Resources, Project administration, Methodology, Investigation, Funding acquisition, Formal analysis, Data curation, Conceptualization. **Masahiro Isogawa:** Writing – review & editing, Visualization, Validation, Supervision, Resources, Project administration, Methodology, Investigation, Funding acquisition. **Hitoki Nanaura:** Writing – review & editing, Visualization, Validation, Investigation, Data curation. **Hiroyuki Kurakami:** Writing – review & editing, Visualization, Validation, Software, Methodology, Formal analysis, Data curation. **Miyoko Hasebe:** Visualization, Validation, Supervision, Investigation, Funding acquisition, Data curation. **Kaoru Kinugawa:** Investigation. **Takao Kiriyama:** Investigation. **Tesseki Izumi:** Investigation. **Masato Kasahara:** Supervision, Resources, Project administration, Funding acquisition, Conceptualization. **Kazuma Sugie:** Writing – review & editing, Supervision, Project administration, Investigation, Funding acquisition.

## Funding

This study is funded by Sumitomo Pharma Co., Ltd. and Nara Medical University Clinical Research Grant Programme.

The authors report no conflicts of interest related with the present manuscript.

## Declaration of competing interest

The authors declare that they have no known competing financial interests or personal relationships that could have appeared to influence the work reported in this paper.
