# Supplementary material for: Effect of zonisamide on sleep and rapid eye movement sleep behavioral disorders in patients with Parkinson’s disease: A randomized control trial
Source: Clin Park Relat Disord. 2024 Nov 22;11:100285. doi: 10.1016/j.prdoa.2024.100285 (PMC11625216; doi:10.1016/j.prdoa.2024.100285)
Supplement: Supplementary Data 1 [file mmc1.docx]

Supplemental table 1

Change of motor scores in the zonisamide treatment and placebo groups

|  | zonisamide treatment (n=33) | | | | placebo (n=34) | | | |
| --- | --- | --- | --- | --- | --- | --- | --- | --- |
|  | baseline | 28 days | quantity of alteration | p | baseline | 28 days | quantity of alteration | p |
| development of Hoehn-Yahr stage, n |  |  | 1 (3.0) |  |  |  | 5 (14.7) | 0.197 ‡ |
| MDS-UPDRS part 3 | 27.9±15.0 | 16.9±13.3 | −11.0±10.2 | <0.001 | 23.9±13.1 | 23.4±15.0 | −0.6±8.8 | 0.715 |
| MDS-UPDRS part 4 | 4.0, 0 to 15.0 | 4.0, 0 to 12.0 | 0, -9 to 6.0 | 0.124 | 4.0, 0 to 17.0 | 3.5, 0 to 14.0 | 0, -8 to 3.0 | 0.535 † |

MDS-UPDRS: Movement Disorder Society Revision of the Unified PD Rating Scale,

data are reported as mean (standard deviation), median (range), or number (%),

‡ Fisher's exact tests, †Wilcoxon's rank sum test
