# Supplementary material for: Effect of zonisamide on sleep and rapid eye movement sleep behavioral disorders in patients with Parkinson’s disease: A randomized control trial
Source: Clin Park Relat Disord. 2024 Nov 22;11:100285. doi: 10.1016/j.prdoa.2024.100285 (PMC11625216; doi:10.1016/j.prdoa.2024.100285)
Supplement: Supplementary Data 2 [file mmc2.docx]

**Supplemental table 2**

Side effects observed in zonisamide and placebo groups.

| side effect | zonisamide group | | placebo group | | p |
| --- | --- | --- | --- | --- | --- |
|  | n（%） | 95% CI（%） | n（%） | 95% CI（%） |  |
| excessive sleepiness | 1 (2.9) | [0.1, 15.3] | 0 (0.0) | [0.0, 10.0] | 0.493 |
| urine incontinence | 1 (2.9) | [0.1, 15.3] | 0 (0.0) | [0.0, 10.0] | 0.493 |
| nausea | 1 (2.9) | [0.1, 15.3] | 0 (0.0) | [0.0, 10.0] | 0.493 |
| falls due to the primary disease | 1 (2.9) | [0.1, 15.3] | 0 (0.0) | [0.0, 10.0] | 0.493 |
| swelling of the left dorsum of the foot | 0 (0.0) | [0.0, 10.3] | 1 (2.9) | [0.1, 14.9] | 1.000 |

* : Fisher's exact test
